# Intra-Individual Variations in How Insulin Sensitivity Responds to Long-Term Exercise: Predictions by Machine Learning Based on Large-Scale Serum Proteomics

**DOI:** 10.3390/metabo14060335

**Published:** 2024-06-15

**Authors:** Jonas Krag Viken, Thomas Olsen, Christian André Drevon, Marit Hjorth, Kåre Inge Birkeland, Frode Norheim, Sindre Lee-Ødegård

**Affiliations:** 1 Institute of Clinical Medicine, Faculty of Medicine, University of Oslo, 0313 Oslo, Norway; j.k.viken@studmed.uio.no (J.K.V.); k.i.birkeland@medisin.uio.no (K.I.B.); 2 Department of Nutrition, Faculty of Medicine, Institute of Basic Medical Sciences, University of Oslo, 0313 Oslo, Norway; thomas.olsen@medisin.uio.no (T.O.); c.a.drevon@medisin.uio.no (C.A.D.); marit.hjorth@medisin.uio.no (M.H.); frode.norheim@medisin.uio.no (F.N.); 3 Vitas Ltd., Oslo Science Park, 0349 Oslo, Norway; 4 Department of Endocrinology, Morbid Obesity and Preventive Medicine, Oslo University Hospital, 0586 Oslo, Norway

**Keywords:** artificial intelligence, proteomics, exercise, insulin sensitivity

## Abstract

Physical activity is effective for preventing and treating type 2 diabetes, but some individuals do not achieve metabolic benefits from exercise (“non-responders”). We investigated non-responders in terms of insulin sensitivity changes following a 12-week supervised strength and endurance exercise program. We used a hyperinsulinaemic euglycaemic clamp to measure insulin sensitivity among 26 men aged 40–65, categorizing them into non-responders or responders based on their insulin sensitivity change scores. The exercise regimen included VO_2_max, muscle strength, whole-body MRI scans, muscle and fat biopsies, and serum samples. mRNA sequencing was performed on biopsies and Olink proteomics on serum samples. Non-responders showed more visceral and intramuscular fat and signs of dyslipidaemia and low-grade inflammation at baseline and did not improve in insulin sensitivity following exercise, although they showed gains in VO_2_max and muscle strength. Impaired IL6-JAK-STAT3 signalling in non-responders was suggested by serum proteomics analysis, and a baseline serum proteomic machine learning (ML) algorithm predicted insulin sensitivity responses with high accuracy, validated across two independent exercise cohorts. The ML model identified 30 serum proteins that could forecast exercise-induced insulin sensitivity changes.

## 1. Introduction

Type 2 diabetes is reaching pandemic proportions, and is characterized by impaired insulin sensitivity [1]. Physical activity is a cornerstone in the prevention and treatment of type 2 diabetes, largely because physical activity may enhance insulin sensitivity substantially [2]. However, the positive effects of physical activity are frequently concluded from average data across groups, ignoring that a substantial portion of individuals, found in the left extreme of the change score distribution, may not obtain substantial metabolic benefits from physical activity [3,4].

The concept of exercise response variability was proposed several decades ago [5], but studies on individual differences in response to physical activity have traditionally focused on VO_2_max [5]. In the HERITAGE study, many participants did not improve their VO_2_max after long-term exercise [6]. Low VO_2_max is linked to impaired muscle mitochondrial function, insulin resistance, and hyperglycaemia [6]. However, despite VO_2_max being highly related to glucometabolic health [7], the HART-D study found that improved risk factors for type 2 diabetes after physical activity may occur independent of changes in VO_2_max [8]. Hence, VO_2_max might be an incomplete measure of the effects of physical activity on risk factors for type 2 diabetes.

More recent studies have shown that individual variation in response to physical activity is linked to distinct genetic [9], epigenetic [10], and transcriptomic [11] patterns. Two studies identified that impaired myocellular ATP synthesis is associated with insulin sensitivity non-response after moderate exercise for six months [9,12]. Another study identified TGF-beta suppression of mitochondrial function in skeletal muscle as a potential mechanism for insulin sensitivity non-response to exercise [13]. Furthermore, phosphocreatine recovery rate, an indicator for impaired skeletal muscle mitochondrial function, is also markedly reduced in insulin sensitivity non-responders to exercise [10], both in muscle tissue and in isolated primary muscle cells [10]. In addition, impaired mitochondrial biogenesis is also associated with insulin sensitivity non-response to exercise [11]. Studies applying large-scale proteomics have also identified angiogenetic [14] and gut microbiota pro-inflammatory [15] factors, as well as factors affecting the intestinal absorption of glucose [15], as predictors of individualized insulin sensitivity responsiveness after long-term exercise. 

Biological individuality has become a major focus in medical research, including for personalized medicine in diabetes [16]. Machine learning (ML) may be applied to predict individual responses based on large amounts of complex data [17] and to sub-type patients with type 2 diabetes [18]. Furthermore, ML has identified physical performance as the strongest risk factor for mortality in older patients with type 2 diabetes [19] and identified non-responders to metformin treatment using large-scale serum metabolomics [20]. Explainable ML algorithms, such as random forest [21], may also efficiently identify persons who benefit from physical activity [15]. 

In the current exploratory study, we analysed variability in insulin sensitivity responses following 12 weeks of supervised intense strength and endurance exercise. We analysed individual data and both continuous and dichotomized variables, the latter by dividing into two groups based on the bottom and top three quintiles of insulin sensitivity change scores. We then described clinical and molecular characteristics in blood, muscle, and adipose tissue both at baseline and in response to the 12 weeks intervention in relation to insulin sensitivity variability. We also constructed and validated an ML algorithm using two independent cohorts to predict individualized responsiveness to exercise using large-scale serum proteomics. 

## 2. Materials and Methods

**Figure 1:**
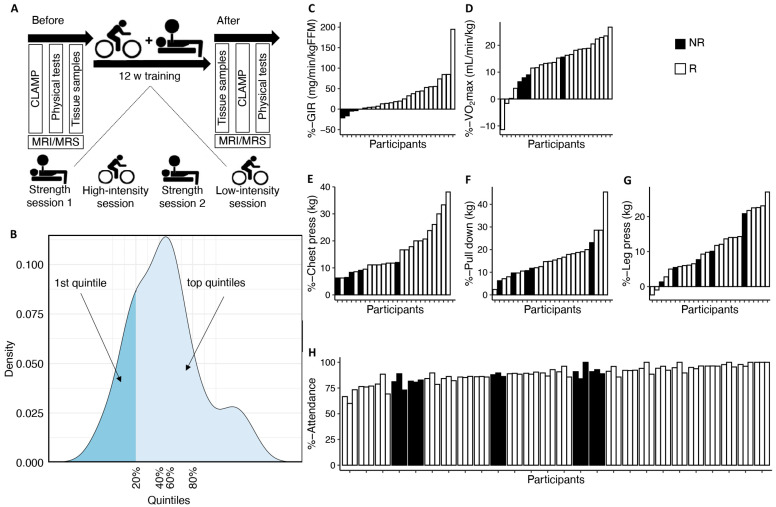
**Effects of the 12-week exercise program on insulin sensitivity and physiological adaptations.** (**A**) A total of 26 men underwent 12 weeks of combined strength and endurance exercise and were phenotyped using a hyperinsulinemic euglycemic clamp, VO_2_max, strength tests, MRI/MRS, and muscle, fat, and blood samplings. (**B**) In the current study, we divided the men into two groups based on their response in insulin sensitivity after the 12-week exercise intervention. The men in the lowest quintile are defined as “non-responders” (NRs) and the men in the top three quintiles as “responders” (Rs). The density curve sums to one and the height of the density curve at a given point represents the relative likelihood of the data points around that value. (**C**–**G**) Waterfall plots showing the responses in main parameters after 12 weeks of exercise. (**H**) Attendance to the intervention for each participant; the left bar is total attendance, the middle bar endurance exercise attendance, and the right bar strength exercise attendance. Panels (**C**–**G**) are % change, calculated as values after the intervention minus the values before intervention, divided by before intervention values and multiplied by 100.

The MyoGlu study has been described in detail previously [2,7,22,23]. Briefly, MyoGlu was a controlled clinical trial (clinicaltrials.gov: NCT01803568) carried out in adherence to the Declaration of Helsinki and received ethical approval from the National Regional Committee for Medical and Health Research Ethics North in Tromsø, Norway (ref. no. 2011/882). All participants provided written informed consent before undergoing any procedures. We included men aged 40–65 years who were healthy and sedentary (<one instance of exercise/week in the previous year). The men underwent 12 weeks of combined strength and endurance training. This 12-week intervention included two weekly sessions of 60 min each for endurance cycling and two sessions of 60 min each for whole-body strength training (Figure 1A).

### 2.1. Insulin Sensitivity and Group Definition 

A euglycemic hyperinsulinemic clamp was performed on each participant after an overnight fast both before and after the 12-week exercise intervention. Insulin sensitivity was calculated as the glucose infusion rate (GIR) during the last 30 min of the clamp relative to magnetic resonance imaging (MRI)-quantified lean mass. In this follow-up study, we grouped the men based on their response in GIR. We then classified two groups based on the lowest vs. the top three quintiles (calculated from the % changes from before to after the intervention) in GIR response (Figure 1B). For simplicity, we termed the men in the lowest quintiles “non-responders” and the men in the top three quintiles “responders”. We supplied these dichotomized analyses with the assessment of individual data, and also analyses using continuous variables without any categorizations.

### 2.2. Cardiovascular Fitness 

VO_2_max tests were performed after a standardized warm-up at a workload similar to the final load of an incremental test in which the relationship between workload (Watt) and oxygen uptake was established. Participants cycled for 1 min followed by a 15 Watt increased workload every 30 s until exhaustion. Test success was based on an O_2_ consumption increase <0.5 mL·kg^−1^·min^−1^ over a 30 Watt increase in workload, respiratory exchange ratio values > 1.10, and blood lactate > 7.0 mmol/L.

### 2.3. Body Composition 

MRI/MRS methods were used to quantify fat and lean mass. The ankle-to-neck MRI protocol included a 3D DIXON acquisition providing water and lipid quantification; data were then analysed using the nordicICE software package v4.0.0 (NordicNeuroLab, Bergen, Norway), and the jMRUI workflow [7]. 

### 2.4. Tissue Samples 

We obtained scWAT, SkM biopsies, and blood samples as described previously [7]. Biopsies were obtained from the periumbilical subcutaneous tissue and from m. vastus lateralis. After sterilization, a local anaesthetic (lidocaine) was injected prior to biopsies. Biopsies were dissected on an ice-cold aluminium plate to remove blood and other contaminants before freezing. For standard serum parameters, measurements were either conducted using standard in-house methods or outsourced to a commercial laboratory (Fürst Laboratories, Oslo, Norway).

### 2.5. mRNA Sequencing

Biopsies were frozen in liquid nitrogen, crushed to powder, transferred into 1 mL of QIAzol Lysis Reagent (Qiagen, Hilden, Germany), and homogenized using TissueRuptor (Qiagen) at full speed for 15 s, twice [7,22]. Total RNA was isolated using the miRNeasy Mini Kit (Qiagen). RNA integrity and concentration were determined using Agilent RNA 6000 Nano Chips on a Bioanalyzer 2100 (Agilent Technologies Inc., Santa Clara, CA, USA). RNA was converted to cDNA using the High-Capacity cDNA Reverse Transcription Kit (Applied Biosystems, Foster, CA, USA). All muscle and scWAT samples were deep-sequenced using the Illumina HiSeq 2000 system with multiplex at the Norwegian Sequencing Centre, University of Oslo. The mean library size was ~50 million unstranded 51 bp single-ended reads. No batch effects were present. A cDNA sequenced read alignment was performed using Tophat v2.0.8, Samtools v0.1.18, and Bowtie v2.1.0 with default settings against the UCSC hg19 annotated transcriptome and genome. Post-alignment quality controls were performed using the Integrative Genome Viewer v2.3 and BED tools v2.19.1. Reads were counted using the intersection strict mode in HTSeq v0.6.1.

### 2.6. Olink Proteomics 

We utilized antibody-based technology (Olink Proteomics AB, Uppsala, Sweden) to conduct profiling of 3072 serum proteins at baseline using the standard work flow as suggested by Olink, and described in detail previously [14]. Briefly, proximity extension assay technology utilizes DNA oligonucleotide-labelled antibody pairs to target and bind proteins, creating a unique DNA barcode through hybridization and extension when antibodies match. This barcode is detected by next-generation sequencing, ensuring high specificity and sensitivity due to the need for precise DNA sequence matching. Values are presented as normalized protein expression (NPX) units on a log_2_ scale.

### 2.7. Analytic Approach

We obtained data from Diaz-Canestro et al. [15], serving as training data for the ML algorithm, including 48 medication-naive overweight/obese men with prediabetes undergoing a 12-week high-intensity interval training (HIIT) regime. Non-responders and responders were defined as obtaining a reduction in HOMA-IR greater than the 2-fold technical error [15] after exercise. Serum proteins were quantified using the Olink Explore 384 cardiometabolic and inflammation panels [15].

Statistical analyses were performed in R. Participants’ baseline characteristics and outcomes were expressed as mean ± SD. Data were assessed for normality using quantile–quantile plots. The difference between groups at baseline and in response to the intervention was evaluated by the independent Welch t test or Kruskal–Wallis test for normal and non-normal variables, respectively. 

Cluster analyses were performed using the R package pheatmap v1.0.12. Pathway analyses were performed using Hallmark pathways from MSigDB [24]. Network analyses were performed using Mergeomics key drives analyses [25]. Differential expression analyses of interaction effects between non-responders and responders in response to 12 weeks of exercise in muscle and adipose tissue, were performed using the R package DESeq2 [26]. For serum proteins, we used the R package lme4 [27] (serum proteins followed a normal distribution in our data). 

The ML algorithm predicting exercise responsiveness was constructed with the R caret [28] package. First, the most informative proteins were selected using a subsample and comparing serum proteins levels between non-responders and responders [15]. Proteins with *p* < 0.1, using a Wilcoxon rank-sum test, were selected, resulting in 32 proteins [15]. Then, several ML algorithms were constructed and compared, and a random forest model was superior to other algorithms, such as generalized linear regression and logistic regression, for predicting insulin sensitivity responses from baseline proteomics data [15]. Because it can be hard to understand how ML algorithms work [29], we have illustrated the concept of a random forest with examples in Supplementary Figure S1. In our current study, we used proteins available both in MyoGlu and in the study of Diaz-Canestro et al. [15], resulting in 30 proteins (two proteins were not available in MyoGlu due to quality filtering). We re-trained the ML algorithm (see Supplementary Materials for raw data and R scripts) on the full data set *(n* = 48) from Diaz-Canestro et al., using 30 proteins [15]. The minor discrepancies in reported area under the receiver operating characteristic curve (AUROC) for the Diaz-Canestro et al. [15] data in this paper compared to what was originally published [15] is due to us re-training the model on the full training data. Originally, Diaz-Canestro et al. [15] used only one cohort, but split this data set in two (one training and one testing set). Furthermore, we only used 30 proteins (originally 32 proteins [15]). Construction of the model involved 5 repetitions of 10-fold cross-validations along with Random Over-Sampling Examples (ROSE) sampling to mitigate the imbalance between non-responders and responders (Supplementary Materials). We then tested it on MyoGlu as a true validation sample. Model performance was evaluated through AUROC. All data and R scripts are freely available as online Supplementary Data.

We also screened for potential serum proteins that might be related to variations in metabolic responses to prolonged exercise. This was conducted by regressing baseline serum protein levels of all 2886 available proteins with the % change score (values after the intervention minus the values before intervention, divided by before intervention values and multiplied by 100) in GIR, VO_2_max, leg press strength, pull down strength, and chest press strength, respectively, using the lm() function in R. Correction for multiple testing was performed by the Benjamini–Hochberg procedure [30,31].

## 3. Results

### 3.1. Phenotypes of Insulin Sensitivity Non-Responders to Prolonged Exercise

We divided the 26 men participating in the MyoGlu study into two groups, the lowest vs. the top three quintiles in GIR response to 12 weeks of combined strength and endurance exercise (Figure 1A,B). Based on individual data, the five men labelled “non-responders” displayed no or a negative change in GIR (Figure 1C). However, all five men did increase their VO_2_max (Figure 1D) and body strength (Figure 1E–G). Non-responders and responders did not differ in attendance to the endurance and strength training sessions during the intervention (Table 1). No correlations were observed between change in GIR and strength training attendance (r = 0.12 and *p* = 0.544), endurance training attendance (r = 0.18 and *p* = 0.370), or total attendance (r = 0.184 and *p* = 0.369). 

Non-responders and responders did not differ in age, body weight, total fat, and fat-free mass, nor in markers of physical fitness (VO_2_max, leg press, pull down, and chest press) pre-training (Table 1). In addition, non-responders displayed similar GIRs to responders pre-training (Table 1). The pre-training characteristics of non-responders included signs of dyslipidaemia, as reflected in lower plasma HDL cholesterol and higher triglyceride concentrations than responders (Table 1). Furthermore, non-responders had more visceral fat mass than responders pre-training (Table 1). Non-responders also displayed higher plasma concentrations of high sensitivity (hs) CRP and IL-6 compared to responders pre-training (Table 1).

Based on group average data, despite no improved insulin sensitivity as measured by the GIR, non-responders did increase their VO_2_max, chest press, leg press, and pull-down strength after 12 weeks of exercise (Table 1). However, their increase in VO_2_max and chest press was significantly lower than in responders (Table 1). 

### 3.2. Skeletal Muscle Characteristics 

**Figure 2:**
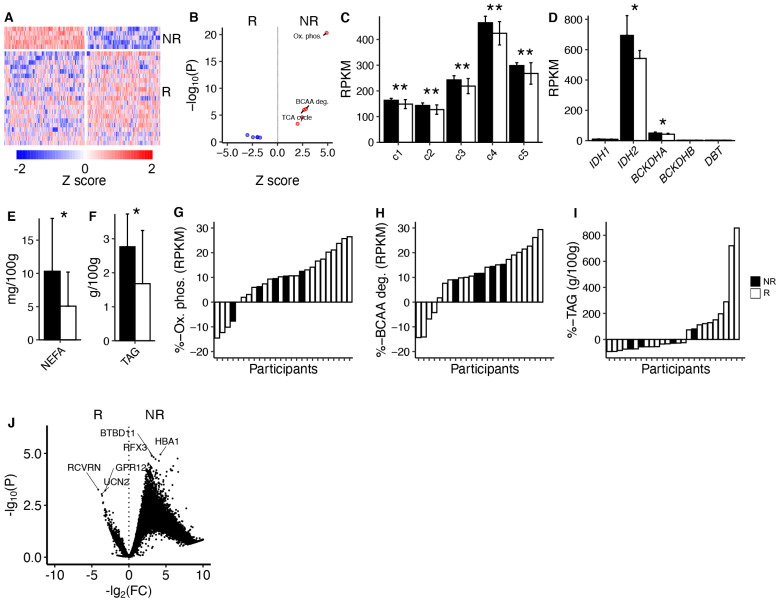
**mRNA expression profiling and metabolic impact of the 12-week exercise program.** (**A**) Clustering of mRNA levels in skeletal muscle between responders and non-responders at baseline. Each row is one person, and the white horizontal line indicates non-responders and responders. Blue colour indicates lower and red colour higher mRNA levels. The white vertical line divides two main clusters, and each column is one mRNA. (**B**) Pathway analysis of the mRNA clusters from A associated with responders and non-responders. (**C**) Mean mRNA levels related to complex 1–5 in the electron transport chain/oxidative phosphorylation. (**D**) mRNA levels related to key enzymes in the BCAA degradation pathway. (**E**,**F**) Muscle NEFA and TAG content. (**G**–**I**) Waterfall plots of responses in mean mRNA levels in electron transport chain/oxidative phosphorylation and BCAA degradation, and muscle TAG content. (**J**) Volcano plot of mRNA changes in muscle in responders vs. non-responders in response to 12 weeks of exercise. NR = non-responder; R = responder. * *p* < 0.05 and ** *p* < 0.01.

Responders and non-responders showed clear differences in global gene expression pre-training, as indicated by cluster analysis of mRNA levels (Figure 2A). Pathway analysis indicated that oxidative phosphorylation, tricarboxylic acid cycle (TCA), and branched-chain amino acid (BCAA) degradation were increased in non-responders pre-training (Figure 2B). Mean mRNA levels, associated with complex 1–5 in the electron transport chain, and mRNA levels of key enzymes in TCA and BCAA degradation were all increased in non-responders pre-training (Figure 2C,D). Non-responders also exhibited elevated levels of muscle non-esterified fatty acids (NEFAs) and triacylglycerol (TAG) pre-training (Figure 2E,F). However, we did not detect any significant difference between non-responders and responders in response to 12 weeks of exercise for mRNA levels associated with oxidative phosphorylation or the TCA cycle, nor in muscle TAG levels (Figure 2G–I). Furthermore, we did not detect any global differences in mRNA responses to the intervention between non-responders and responders after correction for multiple testing (Figure 2J). Please see Supplementary Figure S2 and Supplementary Table S1 for details. 

### 3.3. Adipose Tissue Characteristics 

**Figure 3:**
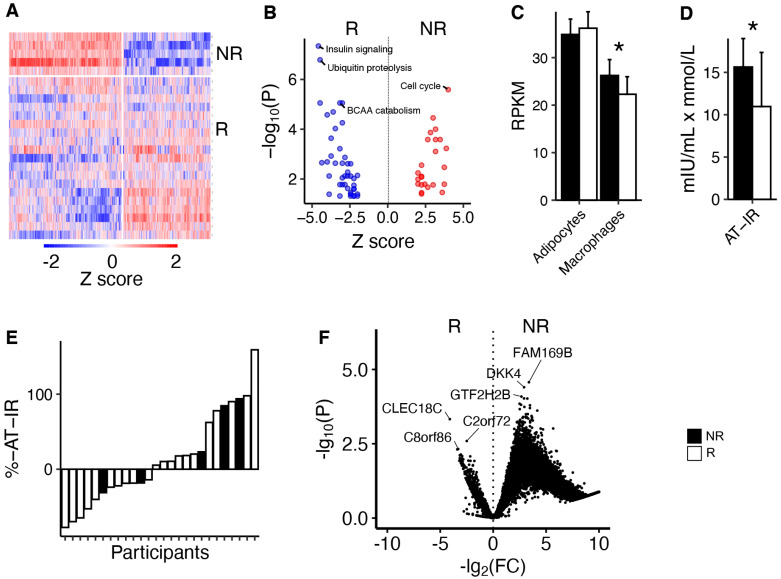
**Adipose tissue adaptions to the 12-week exercise program.** (**A**) Clustering of mRNA levels in adipose tissue between responders and non-responders at baseline. Each row is one person, and the white horizontal line indicates non-responders and responders. Blue colour indicates lower and red colour higher mRNA levels. The white vertical line divides two main clusters, and each column is one mRNA. (**B**) Pathway analysis of the mRNA clusters from A associated with responders and non-responders. (**C**) Mean mRNA levels of adipocyte and macrophage markers. (**D**) The adipose tissue insulin resistance index (product of plasma insulin and NEFA levels). (**E**) Waterfall plot of response in the adipose tissue insulin resistance index. (**F**) Volcano plot of mRNA changes in adipose tissue in responders vs. non-responders in response to 12 weeks of exercise. * *p* < 0.05. NR = non-responder; R = responder.

Responders and non-responders showed clear differences in global gene expression pre-training, as indicated by cluster analysis of mRNA levels (Figure 3A). Pathway analysis indicated that insulin signalling and BCAA catabolism were impaired in non-responders compared to responders pre-training (Figure 3B). Average levels of mRNA markers of adipose tissue macrophages indicated increased numbers in non-responders compared to responders (Figure 3C). Non-responders also exhibited elevated adipose tissue insulin resistance (AT-IR) scores pre-training compared to responders (Figure 3D). There were no differences between non-responders and responders in responses to 12 weeks of exercise in AT-IR or global mRNA analysis after correction for multiple testing (Figure 3E,F). Please see Supplementary Table S2 for details. 

### 3.4. An ML Algorithm to Predict Variations in Insulin Sensitivity Responses by Serum Proteomics

**Figure 4:**
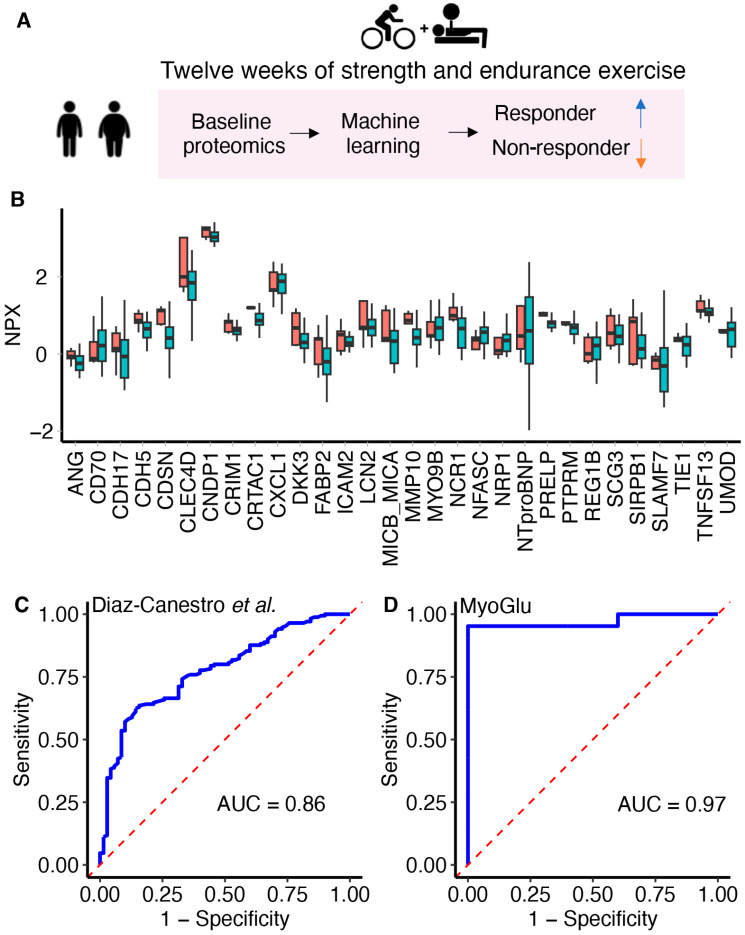
**Predicting exercise-induced insulin sensitivity response using an ML trained on protein levels at baseline.** (**A**) A baseline serum proteomic ML algorithm predicted responsiveness in insulin sensitivity. (**B**) The serum proteins used by the ML algorithm in responders (green) and non-responders (red) in MyoGlu at baseline. (**C**,**D**) The receiver operating characteristic (ROC) curves and area under curve (AUC) of the proteomics-based ML algorithm for the discrimination between non-responders and responders in the training study by Diaz-Canestro et al. [15] (*n* = 48) and in the validation study MyoGlu (*n* = 26). NPX, normalized protein expression; AUROC, area under the receiver operating characteristic curve. The red stapled lines represent the performance of a random classifier, which has no discrimination ability. The blue lines shows the trade-off between true positive rate and false positive rate at various thresholds, and the closer the curve follows the left-hand border and then the top border, the better the model.

Given the importance of circulating proteins as potential mediators of exercise-induced insulin sensitivity, we developed an ML (random forest) algorithm (Figure 4A) based on 30 baseline serum proteins (Figure 4B) to predict insulin sensitivity responses following the 12-week exercise intervention. We trained the model using data from Diaz-Canestro et al. [15], raw data can be found in the Supplementary Materials, and achieved an area under the receiver operating characteristic curve (AUROC) of 0.86 (Figure 4C). The performance of this model was then evaluated in MyoGlu as an external validation cohort and achieved an AUROC value of 0.97 for the discrimination between responders and non-responders (Figure 4D). Corneodesmosin (CDSN), cysteine-rich with EGF-like domains 1 (CRIM1), and neuropilin 1 (NRP1) were the top three proteins in terms of variable importance (Supplementary Figure S3). Average serum levels of CDSN, cartilage acidic protein 1 (CRTAC1), natural cytotoxicity triggering receptor 1 (NCR1), and proline/arginine-rich end leucine-rich repeat protein (PRELP) differed significantly between responders and non-responders at baseline (Kruskal–Wallis test *p* < 0.05) (Figure 4B).

### 3.5. Explorative Analyses of Serum Proteins Predicting Exercise Responses

We also performed analyses using the change in GIR, VO_2_max, chest press, pull down, and leg press as continuous variables (Supplementary Figure S4A–E). None of these analyses reached statistical significance after correction for multiple testing, but several showed nominal associations (Supplementary Tables S3–S7). Here follows the top three positively and negatively associated proteins with each outcome. For the GIR, high baseline levels of formin-like 1 (FMNL1), signal transducing adaptor molecule (STAM), and chymotrypsin c (CTRC) were associated with a larger increase after exercise, whereas high levels of EGF-containing fibulin-like extracellular matrix protein 1 (EFEMP1), collagen type VI alpha 3 chain (COL6A3), and delta-like non-canonical notch ligand 1 (DLK1) were associated with a lower response to exercise (Supplementary Figure S4A). For VO_2_max, LysM domain containing 3 (LYSMD3), mediator of DNA damage checkpoint 1 (MDM1), and G protein-coupled receptor 101 (GPR101) were indicators of a larger increase, whereas asialoglycoprotein receptor 2 (ASGR2), jun proto-oncogene (JUN), and pyruvate dehydrogenase phosphatase catalytic subunit 1 (PDP1) were associated with lower response (Supplementary Figure S4B). For chest press strength, dickkopf WNT signaling pathway inhibitor 1 (DKK1), inositol polyphosphate-1-phosphatase (INPP1), and CAMP responsive element binding protein 3 (CREB3) indicated a higher response, whereas carboxypeptidase X M14 family member 2 (CPXM2), capping actin protein, gelsolin like (CAPG), and integrin subunit alpha V (ITGAV) were associated with a lower response (Supplementary Figure S4C). For pull down strength, myosin heavy chain 7B (MYH7B), nicotinamide riboside kinase 2 (NMRK2), and CD3e molecule (CD3E) were associated with a higher response, whereas C-C motif chemokine ligand 18 (CCL18), polypeptide N-acetylgalactosaminyltransferase 2 (GALNT2), and leukocyte immunoglobulin like receptor B5 (LILRB5) were linked to a lower response (Supplementary Figure S4D). For leg press strength, signal regulatory protein beta 1 (SIRPB1), family with sequence similarity 171 member B (FAM171B), and inosine triphosphatase (ITPA) were linked to a higher response, whereas deleted in colorectal carcinoma (DCC), SRY-box transcription factor 9 (SOX9), and CUB and zona pellucida-like domains 1 (CUZD1) indicated a lower response (Supplementary Figure S4E).

### 3.6. Serum Proteomics in Response to 12 Weeks of Exercise

**Figure 5:**
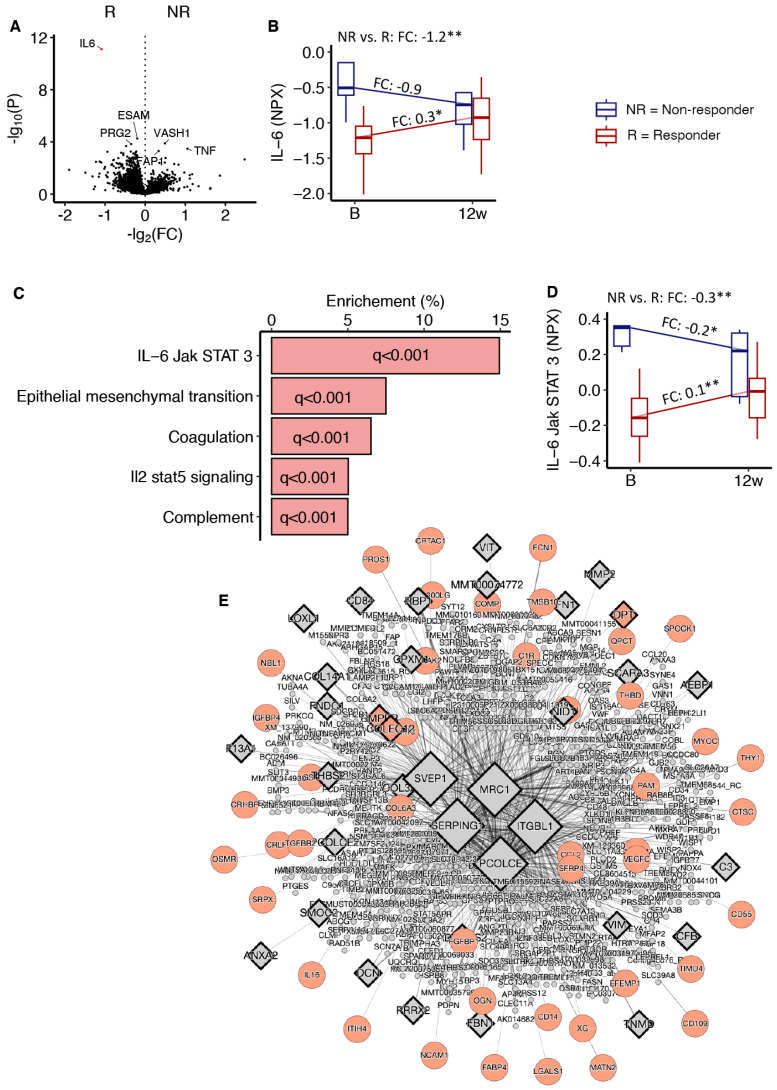
**Proteomic alterations and signalling pathway activation after the 12-week exercise program.** (**A**) A volcano plot of serum protein changes in responders vs. non-responders in response to 12 weeks of exercise intervention. Red indicates corrected *p*-value < 0.05 (only one protein: IL-6). (**B**) Serum IL-6 levels. FC = log_2_(fold-change). (**C**) mRNAs from A with uncorrected *p* < 0.05 subjected to pathway analysis. The pathways enrichment *p*-values were then corrected for multiple testing (q values). (**D**) The median protein levels related to IL-6 Jak/STAT 3 signalling in non-responders (blue) and responders (red). * *p* < 0.05 and ** *p* < 0.01. (**E**) mRNAs from A with uncorrected *p* < 0.05 subjected to network analysis. The diamonds indicate key drivers. Orange circles represent the proteins subjected to analysis and also in the network. Grey circles are proteins in the network but not in the protein set. NR = non-responder; R = responder.

A comparison of the change in serum protein levels in response to the exercise intervention between the two groups revealed a lower response in IL-6 levels in non-responders compared to responders (Figure 5A). Whereas serum IL-6 levels increased in responders, they decreased in non-responders, and the time-by-group interaction effect was significant (Figure 5B). The correlation between baseline serum IL-6 levels quantified with Olink or enzyme-linked immunosorbent assay (ELISA) was r = 0.94, *p* = 2.2 × 10^−11^. Using the top 500 serum proteins from Figure 5A with nominal *p* < 0.05 (Supplementary Table S8) we found evidence of impaired IL-6 Janus kinase (JAK)/ signal transducer and activator of transcription (STAT) 3 in responders compared to responders after intervention (Figure 5B). The median serum protein levels related to IL-6 JAK/STAT3 signalling decreased in non-responders and increased in responders (Figure 5C). The time-by-group interaction effect was also significant (Figure 5C). Network analysis, of the same proteins, suggested an impaired response in proteins associated with tissue and vessel remodelling, such as an impaired response in serum CD300LG levels (Figure 5D). Please see Supplementary Figure S5 for details on the pathway analysis. 

## 4. Discussion

The main finding in our exploratory study was the discovery of distinct characteristics of persons in the lowest quintile (“non-responders”) of insulin sensitivity responses to an exercise intervention. These men had more visceral and intramuscular fat, and signs of dyslipidaemia, low-grade inflammation, and adipose tissue insulin resistance prior to undertaking intense exercise for 12 weeks. Despite an impaired response in insulin sensitivity, these non-responders improved their VO_2_max and muscle strength, although the increase in VO_2_max and chest press strength were significantly lower compared to responders. Large-scale serum proteomics identified impaired IL-6 JAK STAT3 signalling in non-responders in response to the intervention. Furthermore, serum levels of 30 proteins at baseline may predict personalized insulin sensitivity responses with high precision and reproducibility using an ML algorithm. 

Different factors may influence the response to lifestyle interventions in preventing diabetes, such as genetics, epigenetics, and physiological states [32]. We observed that elevated baseline levels of adipose tissue macrophages and insulin resistance, liver fat, and muscle fat seemed to be associated with an attenuated metabolic improvement in response to lifestyle changes [33]. These observations are interesting because they reflect known mechanisms that blunt insulin signalling [1,2]. Adipose tissue insulin resistance is associated with macrophage infiltration and increased lipolysis [1,2]. In turn, increased levels of NEFA are then deposited in liver and muscle and may lead to accumulation of lipid intermediates such as sn1,2-DAG and the activation of PKC δ/ε, which blocks the insulin receptor and thus GLUT4 translocation [1,2]. Hence, persons that display signs of ectopic lipid deposition may be at risk of not obtaining improved insulin sensitivity after exercise [1,2]. 

It should be noted that whereas VO_2_max is closely associated with insulin sensitivity in most individuals [7], the response in VO_2_max to prolonged exercise is primarily influenced by haematological and cardiac adaptations [34,35]. On the other hand, adaptations in muscle and adipose tissues play a significant role in improved insulin sensitivity [2]. Hence, the men in our study with a diminished response in insulin sensitivity may not experience adequate metabolic improvements in muscle and adipose tissue, despite improved haematological and cardiac functions to improve VO_2_max. Our data are in line with observations from the HART-D study demonstrating that enhanced metabolism following exercise intervention may occur regardless of changes in VO_2_max [8].

We also note that our intervention included strength exercise, which is generally much less studied than endurance exercise. Despite observing some men with an impaired response in insulin sensitivity in our study, they improved their muscle strength, which is also an important factor for health and longevity, such as for lower future cancer mortality [36] and diabetes [37].

Circulating proteins are promising predictors of exercise-induced metabolic outcomes, as they can influence biological processes and be measured in a standard blood test. In our data, baseline serum protein levels effectively distinguished between insulin sensitivity non-responders and responders using an ML algorithm constructed and validated in two independent data sets. Our results are in line with a growing body of research indicating that ML is an efficient tool to improve risk stratification and treatment responses in type 2 diabetes [18,19,20]. It is tempting to speculate that our ML algorithm could be used in future studies to a priori define individuals who may not improve their insulin sensitivity in response to exercise, allowing assignment of other interventions, such as weight loss by diet and/or drugs, e.g., to test for non-inferiority against the group assigned to exercise in terms of improved insulin sensitivity. These types of studies may provide more personalized interventions for diabetes.

The proteins identified with the ML algorithm may be biologically interesting. Most of the proteins have known functions in tissue remodelling and the extracellular matrix, such as matrix metallopeptidase 10 (MMP10), Cadherin 17 (CDH17), C-Type Lectin Domain Family 4 Member D (CLEC4D), and many more (Figure 4). We have previously shown that the extracellular matrix is important for exercise adaptations and improved insulin sensitivity [38]. Many of the identified proteins may also be related to different tissues, such as muscle (myosin 6; MYO6B), cartilage (cartilage acidic protein 1; CRTAC1), bone (dickkopf WNT signaling pathway inhibitor 3; DKK3), kidneys (uromodulin; URO), nerves (neurofascin; NFASC), and the pancreas (regenerating family member 1 beta; REG1B), but also skin (lipocalin 2; LCN2, and corneodesmosin; CDSN). Some of the identified proteins are related to angiogenesis, such as angiogenine (ANG), N’neuropilin 1 (NRP1), and tyrosine kinase with immunoglobulin-like and EGF-like domains 1 (TIE1), which is known to be important for insulin sensitivity [14]. Furthermore, fatty acid binding protein 2 (FABP2) is related to lipid metabolism, whereas the N-terminal prohormone of brain natriuretic peptide (NTproBNP) is a well-known marker of heart failure, but less known for its role in insulin sensitivity [39].

Furthermore, our serum proteomic analyses indicated that a lack of increased JAK/STAT3 signalling following 12 weeks of exercise may explain some of the blunted responses in insulin sensitivity in non-responders [40]. This pathway may act as a mediator for important cytokines related to muscle metabolism, such as the IL-6 cytokine family [40]. IL-6/JAK/STAT3 signalling is related to several muscle functions, such as remodelling and angiogenesis [40], which are factors that are important for insulin sensitivity [38]. In addition, the serum proteomic network analysis also implied a blunted response in tissue and blood vessel remodelling in non-responders after exercise, including proteins such as CD300LG. Interestingly, serum CD300LG levels seem causal for glucose homeostasis based on data from the UK biobank, mice knock-out models, and Mendelian randomization analyses [14]. Both serum IL-6 levels and levels of other proteins in JAK/STAT3 signalling were elevated in non-responders vs. responders at baseline, which might seem counter intuitive. However, IL-6/JAK/STAT3 signalling is also strongly related to inflammation [40,41]. Hence, we think that baseline serum levels of these proteins reflect dysmetabolic low-grade inflammation, whereas the change in serum levels of these proteins after exercise reflects adaptations (or lack thereof) in muscle.

The main limitations of our study are the small sample size and lack of data on women. Furthermore, we trained the participants for three months, and cannot exclude the possibility that the non-responders would respond to a longer training period. In addition, a larger study might be able to predict the exact numerical response in insulin sensitivity, not just categorize into response or non-response. We based our analyses on the Olink technique, but validation of the protein-binding specificity of the Olink antibodies using, e.g., LC-MS/MS would have strengthened our findings. Many of our results are also exploratory, such as the pre-training differences between non-responders and responders, and the associations between serum proteins at baseline and subsequent changes in insulin sensitivity and physical fitness after intervention. Hence, these results should only be regarded as suggestive and serve as a basis for future larger studies, including both sexes. The strength of our study is that we have a well-performed and highly controlled exercise intervention. In addition, a common critique of ML algorithms is that they often lack reproducibility. We used two independent exercise cohorts and demonstrated impressive reproducibility despite different exercise regimes, disease status, ethnicity, and methodologies. Serum proteomics together with ML seem promising to predict the individual effects of exercise.

## 5. Conclusions

Improved insulin sensitivity after prolonged exercise may depend on initial levels of, e.g., visceral adipose tissue, as a marker of several metabolic abnormalities impairing insulin sensitivity. Furthermore, IL-6 JAK STAT3 signalling may be important to obtain exercise-induced insulin sensitivity. We also observed that a baseline proteomic signature may predict individual metabolic responses to prolonged exercise using ML. Our findings may facilitate clinical implementation of personalized interventions to prevent diabetes.

## Figures and Tables

**Table 1:** Pre-training data and changes after 12 weeks of exercise intervention.

	Pre-Training		Post-Training (Δ)	
	Non-Responders	Responders	Non-Responders	Responders
Sex (m/f)	5/0	21/0		
Caucasian race	5	21		
Age (years)	55.2 (3.9)	50.2 (6.8)		
Strength attendance (%)	86.2 (9.8)	87.9 (8.3)		
Endurance attendance (%)	87.2 (4.8)	90.6 (9.4)		
Total attendance (%)	86.5 (4.8)	89.3 (8.0)		
HbA1c (mmol/mol)	38 (2.2)	34 (4.4)	N.A.	N.A.
HbA1c (%)	5.6 (0.2)	5.3 (0.4)	N.A.	N.A.
GIR (mg/kgFFM/min)	11.7 (2.5)	13.9 (5.4)	−1.2 (1.4) A	4.5 (3.3) * A
Fasting plasma glucose (mmol/L)	5.6 (0.7)	5.6 (0.5)	0.2 (0.2)	0.1 (0.3)
Fasting insulin (pmol/mL)	65.3 (31.6)	48.7 (25.0)	25.5 (35.1)	1.3 (20.0)
Fasting C-peptide (pmol/mL)	906.8 (399.5)	725.6 (213.5)	127.4 (315.7)	15.0 (163.9)
VO_2_max (mL/kg/min)	38.9 (6.2)	41.0 (5.8)	2.9 (2.2) * A	5.8 (3.5) * A
Chest press (kg)	67.0 (11.1)	67.1 (16.1)	5.5 (1.1) * A	11.4 (4.9) * A
Pull down (kg)	75.5 (8.0)	71.4 (13.7)	9.0 (3.8) *	11.3 (5.2) *
Leg press (kg)	247.0 (36.0)	218.7 (41.5)	23.0 (17.6) *	24.3 (15.9) *
Total cholesterol (mmol/L)	5.4 (0.4)	5.3 (0.7)	0.0 (0.4)	0.0 (0.6)
HDL-C (mmol/L)	1.1 (0.2) A	1.4 (0.3) A	0.1 (0.1)	0.0 (0.2)
LDL-C (mmol/L)	3.4 (0.5)	3.3 (0.6)	0.1 (0.5)	−0.1 (0.4)
Triglycerides (mmol/L)	3.0 (1.6) A	1.6 (0.7) A	−0.8 (2.1)	0.1 (0.9)
Plasma free fatty acids (mmol/L)	0.3 (0.1)	0.2 (0.1)	−0.1 (0.1)	0.0 (0.1)
Body weight (kg)	92.5 (13.2)	85.6 (12.3)	−1.0 (2.5)	−1.0 (2.0) *
Fat mass (L)	42.4 (10.7)	37.4 (9.1)	−3.2 (2.6) *	−2.8 (2.2) *
Fat free mass (L)	38.9 (5.3)	36.9 (4.4)	2.6 (1.6) *	2.0 (1.0) *
SAT (AU)	8034.6 (51.9)	6848.1 (50.3)	−427.0 (22.6)	−582.4 (24.7)
IAAT (AU)	4411.6 (38.3) A	2886.0 (40.1) A	−677.8 (24.5) *	−556.1 (19.4) *
Plasma leptin (ng/mL)	15.5 (8.3)	11.3 (5.8)	−1.8 (2.3)	−3.2 (2.8) *
Plasma adiponectin (ng/mL)	45.5 (15.2)	48.4 (21.0)	−6.8 (8.1) A	−1.6 (3.0) * A
Plasma CRP (ng/mL)	3.9 (3.5) A	1.5 (2.1) A	0.3 (1.0) A	−2.3 (3.7) A

Notes: Data represent the means (SD). Capital letter A indicates a significant difference between responders and non-responders (*p* < 0.05). * *p* < 0.05 pre-training vs. post-training within the same group. For waist–hip ratio measurements, *n* = 15 high responders and *n* = 4 low responders were available. For IL-6, data from *n* = 19 high responders were available. Statistical significance was determined using Welch *t*-tests or Kruskal–Wallis tests. Abbreviations: f, female; m, male; N.A., not available; GIR, glucose infusion rate; FFM, fat-free mass; I, insulin; HDL, high-density lipoproteins; LDL, low-density lipoproteins; C, cholesterol; SAT, subcutaneous adipose tissue; IAAT, intra-abdominal adipose tissue; and AUs, arbitrary units.

## Data Availability

Data are available in the Supplementary Materials or from the corresponding author at a reasonable request.

## Supplementary Materials

The following supporting information can be downloaded at: https://www.mdpi.com/article/10.3390/metabo14060335/s1, Figure S1: Explanation of random forest; Figure S2: Muscle mRNA related to oxidative phosphorylation at baseline; Figure S3: Variable importance from the random forest model; Figure S4: Baseline protein level vs. change score for VO_2_max and strength parameters; Figure S5: Pathway analysis of proteins associated with an impaired response to exercise; Table S1: Differences in muscle mRNA responses to 12 weeks of exercise between responders and non-responders; Table S2: Differences in muscle mRNA responses to 12 weeks of exercise between responders and non-responders; Table S3: Baseline protein levels and associations with the change in glucose infusion rate following 12 weeks of exercise; Table S4: Baseline protein levels and associations with the change in VO_2_max following 12 weeks of exercise; Table S5: Baseline protein levels and associations with the change in chest press strength following 12 weeks of exercise. Table S6: Baseline protein levels and associations with the change in pull down strength following 12 weeks of exercise: Table S7: Baseline protein levels and associations with the change in leg press strength following 12 weeks of exercise; Table S8: Differences in serum proteomics to 12 weeks of exercise between responders and non-responders.
