# Supplementary material for: Intra-Individual Variations in How Insulin Sensitivity Responds to Long-Term Exercise: Predictions by Machine Learning Based on Large-Scale Serum Proteomics
Source: Metabolites. 2024 Jun 15;14(6):335. doi: 10.3390/metabo14060335 (PMC11206077; doi:10.3390/metabo14060335)
Supplement: Supplementary file 1 [file metabolites-14-00335-s001.zip › Supplementary_Figures.pdf]

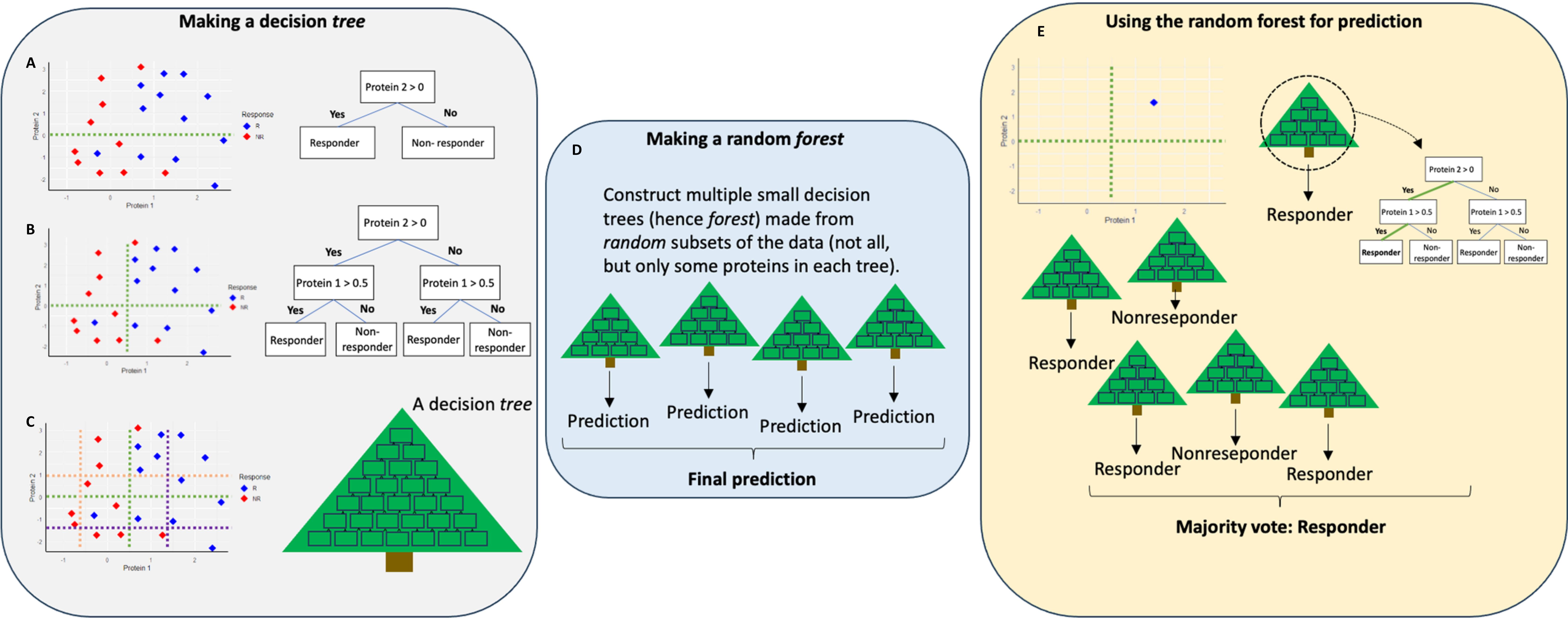

Supplementary Figure S1. A simplified explanation of the random forest model applied to protein-level data for the classification of participants into responders (R) and non-responders (NR). Our model was trained on baseline plasma protein levels alongside known response outcomes in one data set, and then tested on another data set. (A) To illustrate the concept, first a decision tree is made. In this example decisions are made based on a threshold for protein levels for "Protein 2". The horizontal line indicates the cut-off value where we decide that if a person has a protein level above this cut-off the person is a responder, but if it is below the cut-off, the person is non-responder. (B) The decision trees can me more intricate, so in this example decisions are made based on thresholds for two proteins; "Protein 1" and "Protein 2". (C) In reality, a decision tree is usually very complex and includes several proteins. (D) Research has shown that instead of using only one large decision tree, it is usually better to make many smaller decision trees. Meaning that many decision trees are made from a random subset of proteins. Since the proteins are chosen at random, and we construct many decision tress, this technique is called "random forest". (E) When the random forest is constructed from a data set, the model can be used to make predictions on other data sets. Here, the random forest model processes the protein levels from a new participant with an unknown response outcome. Each decision tree independently classifies the participant as either a responder or non-responder. The outcomes from all decision trees are aggregated, and the classification receiving the majority of votes is assigned as the final predicted response for the participant.

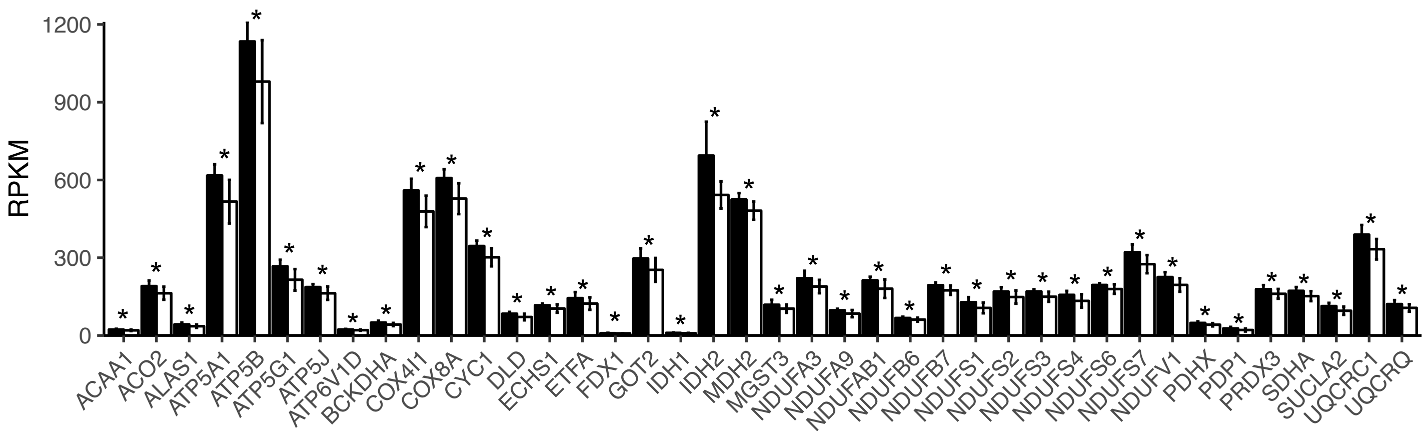

Supplementary Figure S2. Muscle mRNA levels associated with oxidative phosphorylation between non-responders (black) and responders (white) pre-training. *p<0.05. RPKM = Reads per kilobase per million mapped reads,

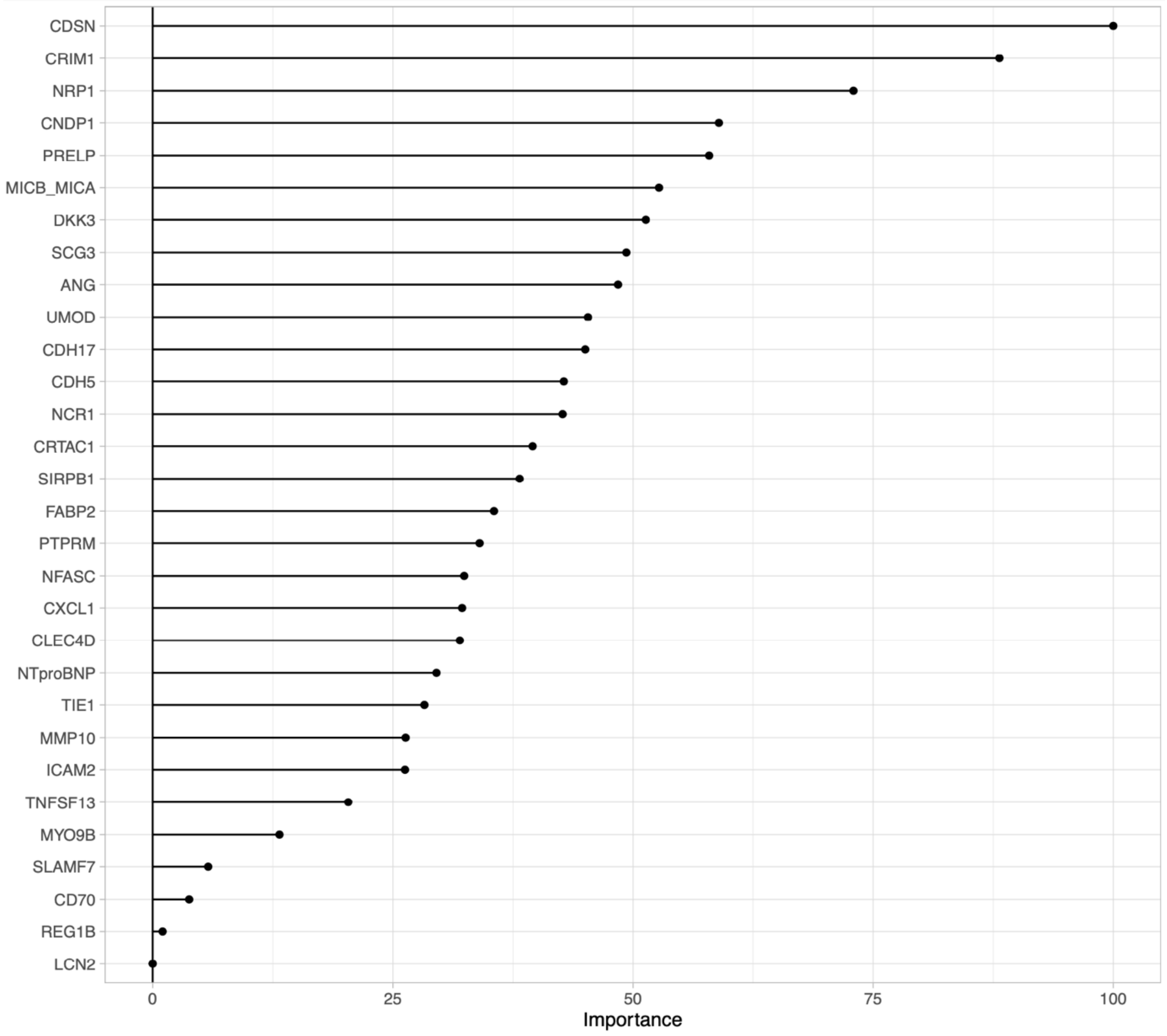

Supplementary Figure S3. Variable importance (VIP) for the proteins used by the AI (random forest) algorithm. VIP refers to a measure that indicates how much each protein contributes to the prediction accuracy of the model. Random forest, being an ensemble learning method that operates by constructing a multitude of decision trees at training time and outputting the class that is the mode of the classes of the individual trees, relies on the contribution of each protein to improve its prediction.

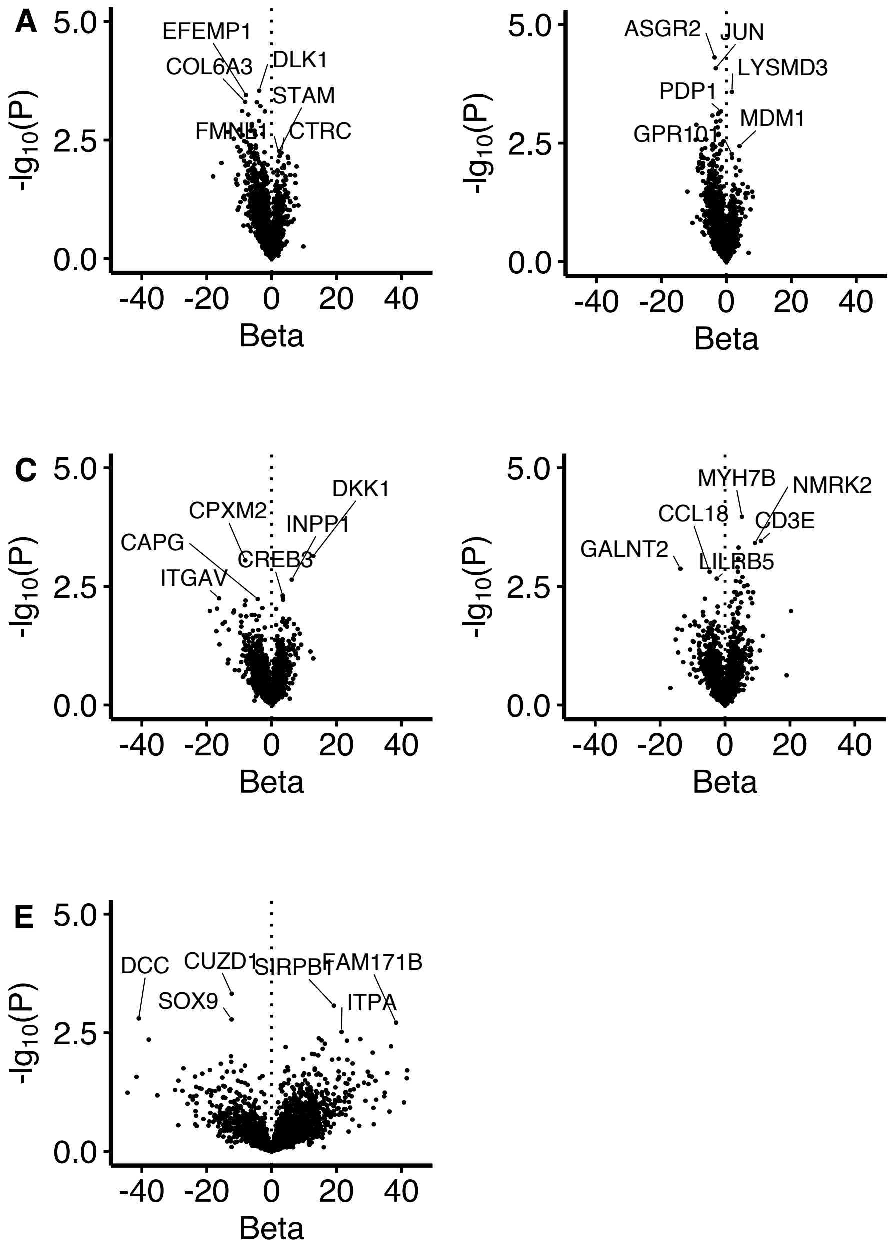

Supplementary Figure S4. Vulcano plots of baseline serum protein levels and associations with change scores in glucose infusion rate (A), VO2max (B), chest press strength (C), pull down strength (D) and leg press strength (E).

Gene Set Name [# Genes (K)]	Description	# Genes in Overlap (k)	k/K	p-value	FDRq-value
HALLMARK_IL6_JAK_STAT3_SIGNALING [87]	Genes up-regulated by IL6 [GeneID=3569] via STAT3 [GeneID=6774], e.g., during acute phase response.	13		$5.33\ e^{-15}$	$2.66\ e^{-13}$
HALLMARK_EPITHELIAL_MESENCHYMAL_TRANSITION [200]	Genes defining epithelial-mesenchymal transition, as in wound healing, fibrosis and metastasis.	15		$1.33\ e^{-12}$	$3.33\ e^{-11}$
HALLMARK_COAGULATION [138]	Genes encoding components of blood coagulation system; also up-regulated in platelets.	9		$1.48\ e^{-7}$	$2.47\ e^{-6}$
HALLMARK_IL2_STAT5_SIGNALING [199]	Genes up-regulated by STAT5 in response to IL2 stimulation.	10		$3.43\ e^{-7}$	$2.99\ e^{-6}$
HALLMARK_COMPLEMENT [200]	Genes encoding components of the complement system, which is part of the innate immune system.	10		$3.59\ e^{-7}$	$2.99\ e^{-6}$
HALLMARK_MYOGENESIS [200]	Genes involved in development of skeletal muscle (myogenesis).	10		$3.59\ e^{-7}$	$2.99\ e^{-6}$
HALLMARK_APOPTOSIS [161]	Genes mediating programmed cell death (apoptosis) by activation of caspases.	9		$5.48\ e^{-7}$	$3.92\ e^{-6}$
HALLMARK_ALLOGRAFT_REJECTION [200]	Genes up-regulated during transplant rejection.	9		$3.31\ e^{-6}$	$1.51\ e^{-5}$
HALLMARK_HYPOXIA [200]	Genes up-regulated in response to low oxygen levels (hypoxia).	9		$3.31\ e^{-6}$	$1.51\ e^{-5}$
HALLMARK_INFLAMMATORY_RESPONSE [200]	Genes defining inflammatory response.	9		$3.31\ e^{-6}$	$1.51\ e^{-5}$

Supplementary Figure S5. All pathways associated with serum proteins showing an impaired response to 12 weeks of exercise in non-responders.
